# Optimization of the extraction process for Shenshou Taiyi powder based on Box-Behnken experimental design, standard relation, and FAHP-CRITIC methods

**DOI:** 10.1186/s12906-024-04554-7

**Published:** 2024-07-02

**Authors:** Mengcheng Jiang, Zhidong Qiu, Yuanyuan Diao, Yuwen Shi, Weipeng Liu, Na Li, Ailing Jia

**Affiliations:** College of Pharmacy, Changchun University of Chinese Medicine, Changchun, 130117 Jilin China

**Keywords:** Ancient formulas, Content determination, Optimization of process, Response surface, Fuzzy analytic hierarchy process, CRITIC method

## Abstract

**Background:**

Ancient classic prescription play a crucial role in the preservation and advancement of traditional Chinese medicine (TCM) theories. They represent a significant milestone in the ongoing development and transmission of TCM knowledge and practices and are considered one of the breakthroughs in the development of TCM inheritance. In the process of developing ancient classic prescriptions, many problems may still arise in ensuring quality consistency between traditional methods and modern production processes, among which the extraction process poses major challenges. This paper introduces a practical approach extracting an ancient classic prescription using a modern extraction process. The technique is demonstrated through the study of the extraction process of Shenshou Taiyi powder (STP).

**Methods:**

This study focuses on optimising the STP extraction process to ensure consistency in the quality of the product obtained through ancient and modern processes using the standard relation and fuzzy analytic hierarchical process (FAHP) and criteria importance through intercriteria correlation (CRITIC) method integrated weights combined with the Box-Behnken response surface test. Using the contents of rosmarinic acid, isoimperatorin, puerarin, as well as the extract yield and fingerprint similarity as evaluation indexes of STP, the Box-Behnken response surface method was employed to examine the varying extraction parameters, including water addition ratio, extraction duration, and number of extractions. The weighted coefficients for each parameter were calculated by combining the benchmark correlation and FAHP-CRITIC method, deriving a comprehensive score.

**Results:**

The optimal extraction process for STP consisted of a two extractions, each using at a tenfold quantity of water, performed for one hour. Process verification across three separate batches yielded a comprehensive score of 94.7, with a relative standard deviation of 0.76%.

**Conclusions:**

The application of the Box-Behnken response surface method combined with standard relation and FAHP-CRITIC approach proved to be stable and feasible for optimising the extraction process of STP.

**Supplementary Information:**

The online version contains supplementary material available at 10.1186/s12906-024-04554-7.

## Background

Shenshou Taiyi powder (STP), was first recorded in ‘*Selected Prescriptions Handed Down for Generations of Physicians*’ by Wang Qiu in the Song Dynasty and consists of eleven herbs, including dried leaves of *Perilla frutescens* (L.) Britt.(Zisuye in Chinese), dried root of *Angelica dahurica.* (Baizhi in Chinese), dried root of *Pueraria lobata* (Willd.) Ohwi. (Gegen in Chinese), dried roots of *Crepis foetida* L.(Shengma in Chinese), dried root of *Ligusticum chuanxiong* Hort. (Chuanxiong in Chinese), dried root of *Cyperus rotundus* L. (Xiangfu in Chinese), dried root of *Paeonia lactiflora* Pall. (Baishao in Chinese), dried peel of ripe *Citrus reticulata* Blanco. (Chenpi in Chinese), pericarp of unripe fruit of *Citrus reticulata* Blanco. (Qingpi in Chinese), dried root of *Glycyrrhiza uralensis* Fisch. (Gancao in Chinese) and fresh root of *Zingiber officinale* Rosc. (Shengjiang in Chinese) [1].

In the process of developing ancient formulas, it is necessary to consult the ancient prescription books, medical books, and other records as a basis for modern formulas, so as to respect the ancient, and at the same time, retain consistency between ancient and modern formulations, the current production methods, and the application of the actual production process. This ensures the development of processes that integrate the salient aspects of both ancient knowledge and modern techniques [2]. At present, orthogonal experimental design, uniform design experimentation, Box-Behnken response surface methodology, and other methods are commonly used in the modernisation of traditional Chinese medicine extraction processes. The evaluation criterion is typically the maximum extraction efficiency of the components of traditional Chinese medicine; however, modern extraction processes do not always meet the requirement of quality consistency between ancient and modern approaches.

Here, we thus started from the concept of quality by design (QbD) [3] to with the objective of ensuring consistency of product quality between ancient and modern samples. We calculated the standard relation (SR) of different index parameters and determined the weighting coefficients of different evaluation indexes through the subjective-objective combination of fuzzy analytic hierarchy process (FAHP) and criteria importance through intercriteria correlation (CRITIC) method to carry out comprehensive scoring. Then, the modern extraction process parameters were screened to ensure quality consistency of the samples optimised by the modern process and the reference samples prepared through the ancient extraction process and to lay a foundation for the subsequent development and application of STP.

## Methods

### Instruments

Five-decimal scales (TG328A, Shanghai Precision Scientific Instrument Co., Shanghai, China); Universal pulveriser (BJ-150, Zhejiang Deqing Baijie Electric Appliance Co., Zheijiang, China); Ultrasonic cleaner (KQ-500B, Kunshan Ultrasonic Instrument Co., Shanghai, China); Rotary Evaporator (RE-52, Shanghai Yarong Biochemistry Instrument Factory, Shanghai, China); Shimadzu High Performance Liquid Chromatograph (LC-15 C, Shimadzu, Kyoto, Japan); Lyophiliser (Epsilon 2–4 LSC plus, Martin Christ Gefriertrocknungsanlagen GmbH, Osterode am Harz, Germany).

### Reagents

Rosmarinic acid, isoimperatorin, and puerarin reference standard substances were provided by Shanghai Yuanye Bio-Technology Co. (Batch No.: Y06A9K67402, M19GB148925, S02M9B54875, Shanghai, China); chromatography-grade acetonitrile and methanol were purchased from Thermo Fisher Scientific (Shanghai, China) Co., Ltd; chromatography-grade phosphoric acid was purchased from Tianjin Guangfu Fine Chemical Research Institute (Tianjin, China); analytical-grade anhydrous ethanol and anhydrous methanol were acquired from Guangdong Guanghua Sci-Tech Co., Ltd (Shantou, China); deionised water was used.

### Herbs

Zisuye (Batch No.: 20210221-01), Baizhi (Batch No.: 20210508-01), Gegen (Batch No.: 20,210,618), Shengma (Batch No.: 20,210,406), Chuanxiong (Batch No.: 20210508-01), Xiangfu (Batch No.: 20,210,423), Baishao (Batch No.: 202,011,218), Chenpi (Batch No.: 20,210,103), Qingpi (Batch No.: 20,180,911), Gancao (Batch No.: 20,210,121) and Shengjiang (Batch No.: 20180714-04) were provided by Jilin North Medicine Herb Processing Co., Jilin, China.

### Preparation of STP reference samples

The preparation process of STP reference samples was determined by analysing relevant literature combined with the prescription of the ancient original records. The preparation process was as follows: The herbs were crushed into coarse powder, after which 8.673 g of each herb was weighed and added to 3150 mL water and 21 g Shengjiang (21 slices; each slice weighed approximately 1 g), and the mixture was decocted to 2520 mL. The decoction was filtered through a 200-mesh double-layer nylon filter cloth, and the aqueous decoction was prepared for the STP reference samples. It was pre-frozen at -20 °C for 12 h and then placed in a freeze dryer at -80 °C for 30 min and freeze-dried for 72 h to obtain lyophilised STP powder as reference samples.

### Determination of fingerprints of Chinese materia medica

*Chromatographic Conditions.* Chromatographic column: Agilent ZORBAX Eclipse Plus C18 (4.6 mm × 250 mm, 5 μm). Mobile phase: acetonitrile (B) − 0.1% phosphoric acid solution (A). Gradient elution for 0 to 15 min: 10% B; for 15 to 30 min: 10–15% B; for 30 to 58 min: 15–21% B; for 58 to 70 min: 21–25% B; for 70 to 80 min: 25–40% B; for 80 to 100 min: 40–55% B; for 100 to 105 min: 55–10% B; for 105 to 120 min: 10% B. Flow rate: 1.0 mL.min^− 1^. Measurement wavelength: 270 nm. Column temperature: 30 °C. Injection volume: 10 µL.

### Preparation of test solution

A sample of 1 g of STP lyophilised powder was weighed, and 25 mL of 75% methanol was added. The mixture was extracted by ultrasonication for 30 min, cooled, and restored to its original volume using 75% methanol. The solution was then filtered through a 0.22 μm microporous filter membrane, and the filtrate obtained was used as test solution.

### Preparation of reference standards solution

Appropriate amounts of rosmarinic acid, isoimperatorin, and puerarin reference standard substances were weighed accurately, and methanol was added to produce reference standard solutions at concentrations of 9.92, 3.96, and 47.2 µg/mL, respectively.

### Determination of the content of indicator components

*Determination of Rosmarinic Acid Content.* A sample of 1 g of STP lyophilised powder was weighed and added to 25 mL 50% ethanol. Ultrasonic extraction was performed for 30 min, after which the mixture was cooled and restored to the original volume using 50% ethanol. The solution was then filtered through a 0.22 μm microporous filter membrane, and the obtained filtrate was the used as the test solution. Chromatographic column: Agilent ZORBAX Eclipse Plus C18 (4.6 mm × 250 mm, 5 μm). Mobile phase: acetonitrile (B) – 0.1% phosphoric acid solution (A). Isocratic elution for 0 to 120 min: 15% B. Flow rate: 1.0 mL/min. Measurement wavelength: 330 nm. Column temperature: 30 °C. Injection volume: 10 µL.

*Determination of Isoimperatorin Content* A sample of 2 g of lyophilised STP powder was weighed and added to 25 mL anhydrous methanol. Ultrasonic extraction was performed for 30 min, after which the mixture was cooled and restored to the original volume using anhydrous methanol. The solution was then filtered through a 0.22 μm microporous filter membrane, and the obtained filtrate was used as the test solution. Chromatographic column: Agilent ZORBAX Eclipse Plus C18 (4.6 mm × 250 mm, 5 μm). Mobile phase: acetonitrile (B) – water (A). Isocratic elution for 0 to 65 min: 42% B. Flow rate: 1.0 mL/min. Measurement wavelength: 300 nm. Column temperature: 30 °C. Injection volume: 10 µL.

*Determination of Puerarin Content.* A sample of 0.1 g of lyophilised STP powder was weighed and added to 50 mL 70% ethanol. Ultrasonic extraction was performed for 30 min, after which the mixture was cooled and restored to the original volume using 70% ethanol. The solution was then filtered through a 0.22 μm microporous filter membrane, and the obtained filtrate was used as the test solution. Chromatographic column: Agilent ZORBAX Eclipse Plus C18 (4.6 mm × 250 mm, 5 μm). Mobile phase: methanol (B) − water (A). Isocratic elution for 0 to 45 min: 21% B. Flow rate: 1.0 mL/min. Measurement wavelength: 271 nm. Column temperature: 30 °C. Injection volume: 10 µL.

### Single-factor investigation experiment

*Effect of Extraction Time.* The prescribed amount of STP herbs were weighed, and a fixed volume of water eight times that of the sample weight was added. Extractions were performed twice for either 0.5, 1, 1.5, or 2 h. The content of rosmarinic acid, isoimperatorin, puerarin, as well as the extract yield were measured to determine the effects of extraction time.

*Effect of Water Addition* The prescribed amount of STP herbs was weighed. The extraction time was fixed at 1 h, and extraction was performed twice. Either 6, 8, 10, or 12 times the volume of water was added, and the rosmarinic acid, isoimperatorin, puerarin and extract yield was determined to examine the effects of water volume used in the extraction process.

### Box-Behnken experimental design

Based on the results of the single-factor investigation experiment, the extraction durations and water volumes resulting in the highest and lowest yields were determined, as well as the high (+ 1) and low (-1) levels of the number of extractions according to the actual production needs and experience, and a centre point (0) level was set. A Box-Behnken experimental design was carried out, and the prescribed amount of each herb was weighed for the experiments. The rosmarinic acid (Y_1_), isoimperatorin (Y_2_) and puerarin (Y_3_) contents as well as the extract yield (Y_4_) were determined. The test solutions of reference sample (S1) of STP and the test solutions of 17 response surface experiments (S2 through S18) were prepared according to the method under “*Preparation of Test Solution*” and were determined according to the method under “*Chromatographic Conditions*”. The reference sample (S1) was used as the reference fingerprint.

## Results

### Analytical method validation

**Fig. 1:**
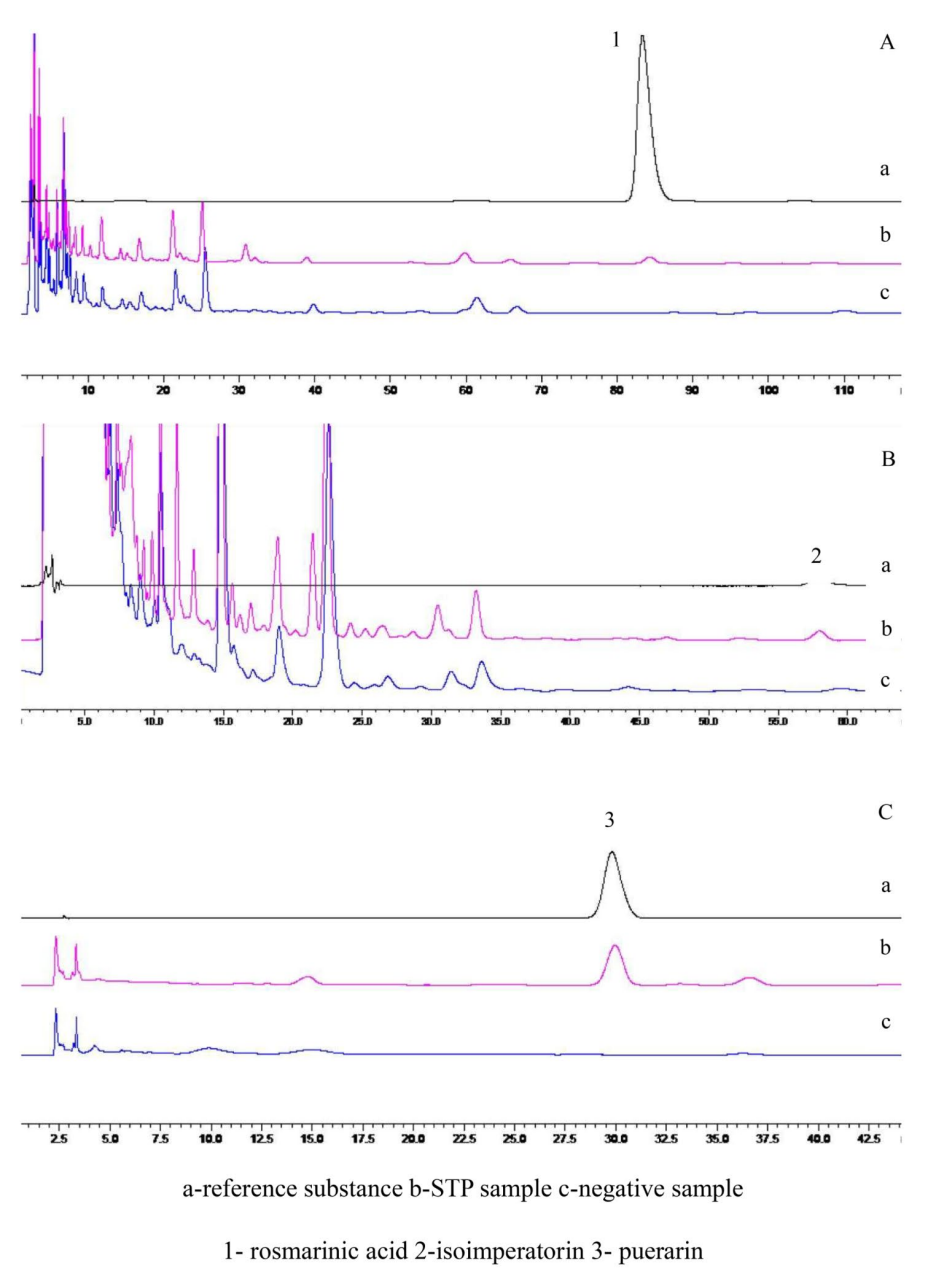
HPLC of specificity determinations of (**A**) rosmarinic acid, (**B**) isoimperatorin, and (**C**) puerarin

The test solution was prepared, and negative test solutions without either Zisuye, Baizhi, or Gegen were prepared. The content was determined according to the content determination method, and the results are shown in Fig. 1. According to the guideline for the validation of analytical methods of the 2020 edition of the *Chinese Pharmacopoeia* (Part IV 9101) [4], linear relation, precision, reproducibility, stability, and sample recovery were examined, and the results are shown in Table 1. The method exhibited good specificity, and all examined indexes were in accordance with the requirements.

**Table 1:** Determination of the content of index components in STP

Indicator components	Linear relation	Linear range(µg/mL)	RSD%			Sample recovery(RSD%)
			Precision	Reproducibility	Stability	
Rosmarinic acid	y = 28397x-39,557 (R² = 0.9998)	4.9–79.3	0.8	1.1	2.5	97.6 (2.4)
Isoimperatorin	y = 30608x-14,984 (R² = 0.9990)	0.2–23.8	1.0	1.0	1.3	101.2 (2.0)
Puerarin	y = 41355x + 26,996 (R² = 0.9996)	3.8–47.2	0.7	0.6	0.5	100.9 (1.3)

### Single-factor investigation experiment

*Effect of Extraction Time.* The rosmarinic acid, isoimperatorin, and extract yields showed an increasing and then decreasing trend; the content of puerarin showed an overall increasing trend with increased extraction time; the isoimperatorin, puerarin, and extract yields were the lowest when the extraction time was 0.5 h. The results showed that the optimal extraction times were those exceeding 0.5 h. For comprehensive consideration, the extraction times with highest and lowest effectiveness were chosen to be 2 and 1 h, respectively, as shown in Table 2.

**Table 2:** Results of extraction time

Extraction time/h	Mass fraction (mg/g)			Extract yield (%)
	Rosmarinic acid	Isoimperatorin	Puerarin	
0.5	1.0101	0.0065	13.6082	20.4
1	1.1950	0.0101	13.9572	23.5
1.5	0.9721	0.0109	14.1734	23.3
2	0.9005	0.0074	14.2169	21.7

*Effect of Water Addition.* The content of rosmarinic acid gradually decreased with increased water volume; the content of isoimperatorin was stable; the content of puerarin showed an increasing and then decreasing trend. The rosmarinic acid content was lowest when the water volume was 12 times the sample weight. For comprehensive consideration, the water volumes with the highest and lowest yields were chosen to be 10 times and 6 times, respectively, as shown in Table 3.

**Table 3:** Results of water addition

Water addition/times of solvent volume	Mass fraction (mg/g)			Extract yield (%)
	Rosmarinic acid	Isoimperatorin	Puerarin	
6	1.3767	0.0097	12.3057	19.8
8	1.2950	0.0101	12.9572	23.5
10	1.2447	0.0105	14.5220	24.4
12	1.1640	0.0100	12.4333	24.1

### Box-Behnken experimental design results

**Fig. 2:**
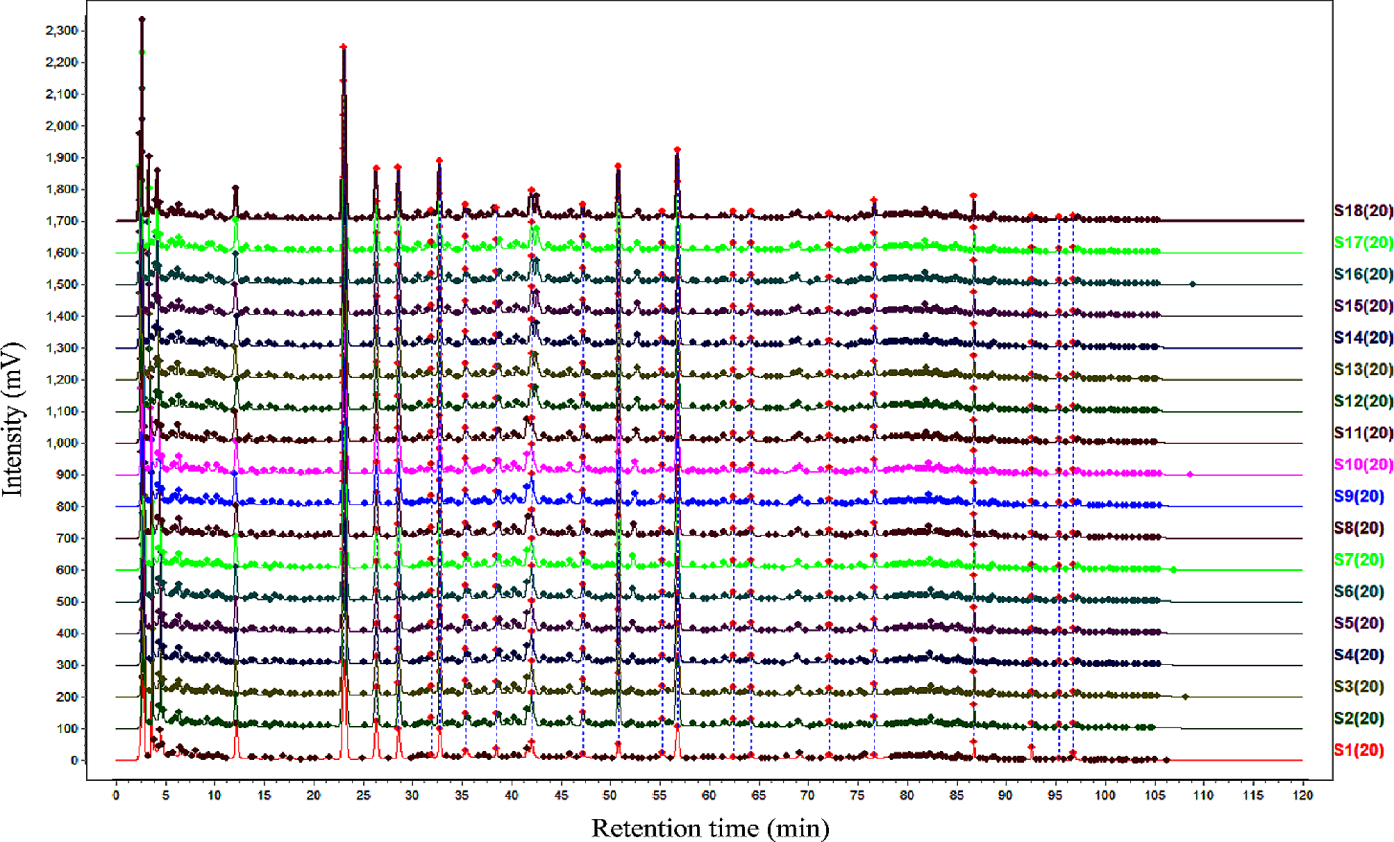
The HPLC fingerprints of reference sample (S1) and response surface test samples (S2–S18)

The experimental design results are shown in Fig. 2. An additional file shows this in more detail (see Additional file 1).

**Fig. 3:**
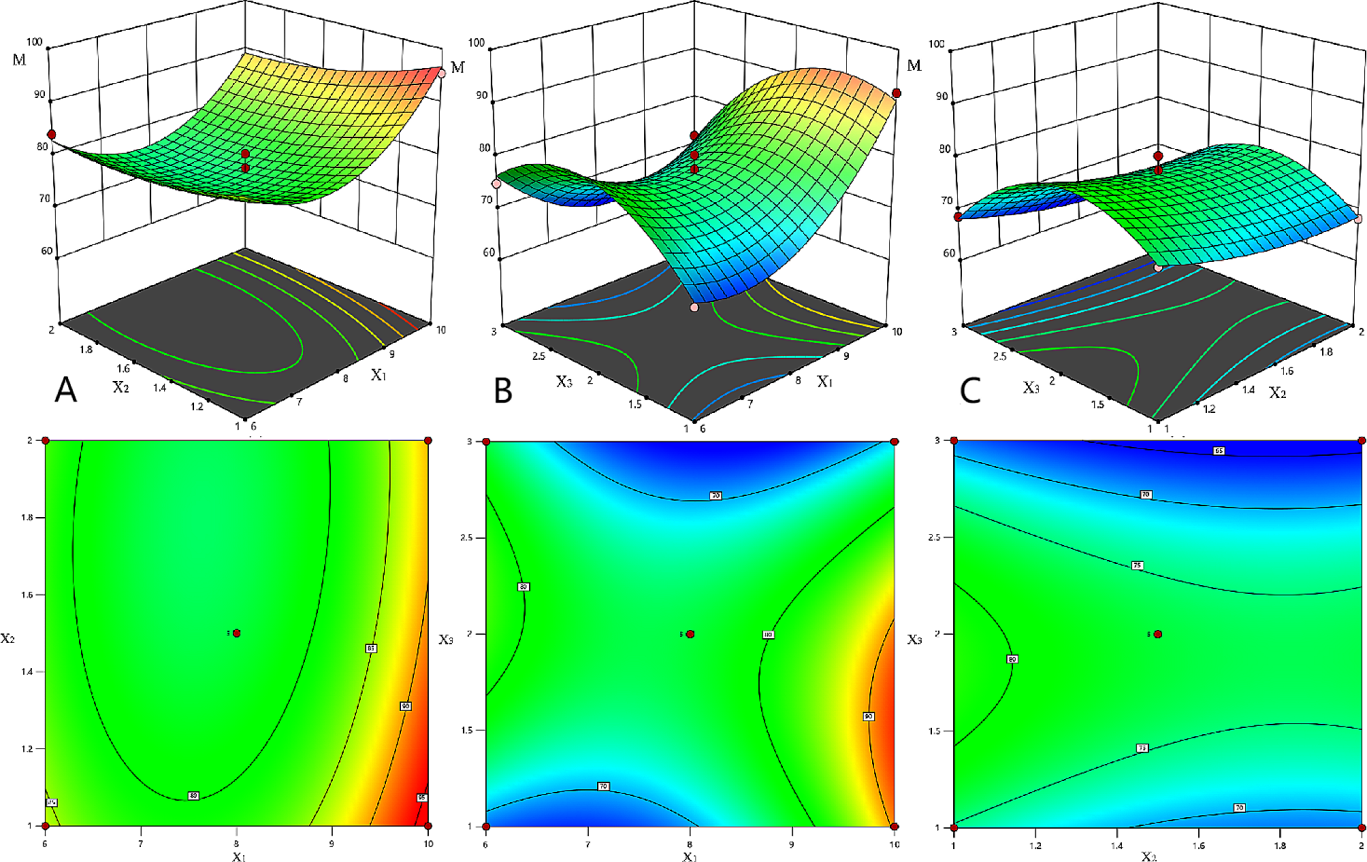
The effect of number of times of water volume and extraction duration (**A**), number of times of water volume and number of extractions (**B**), extraction duration and number of extractions (**C**) on comprehensive score (M)

Design-Expert software was applied to analyse the experimental results, and a multivariate quadratic regression equation model was obtained for the comprehensive score (M) and the number of times of water volume (X_1_), extraction duration (X_2_), and number of extractions (X_3_): M = 142.47-28.591 × _1_-23.15 × _2_ + 64.41 × _3_-1.08 × _1_ × _2_-3.48 × _1_ × _3_ + 0.69 × _2_ × _3_ + 2.46 × _1_^2^ + 8.35 × _2_^2^-10.12 × _3_^2^. Analysis of variance was performed on the model equations, and the results are shown in Table 4, the model of the constructed equations was extremely significant (F _model_ = 30.34, *P* < 0.01); the lack of fit was not significant (*P* > 0.05); the model R_adj_^2^ = 0.9750, R_pred_^2^ = 0.8320, which indicates that the model of this equation is suitable and reliable, with small experimental error and goodness of fit, and it can be used to analyse and predict the STP extraction process. From the P value, X_1_, X_2_, X_3_, X_1_ × _3_, X_1_^2^, X_2_^2^, and X_3_^2^ of the independent variables of the model had a significant effect on the comprehensive score (*P* < 0.01, *P* < 0.05), and X_1_ × _2_ and X_2_ × _3_ in the interaction terms had no significant effect on the composite score (*P* > 0.05). From the F value and the response surface plots (Fig. 3), the order of the effect of each factor on the composite score was X_1_ > X_3_ > X_2_.

**Table 4:** Response surface model ANOVA

Source	Sum of Squares	Degree of freedom	Mean Square	F value	*P* value
Model	1277.9100	9	141.9900	30.3400	< 0.0001**
X_1_	144.5900	1	144.5900	30.9000	0.0009**
X_2_	56.9700	1	56.9700	12.1800	0.0101*
X_3_	64.4100	1	64.4100	13.7600	0.0076**
X_1_ × _2_	4.6300	1	4.6300	0.9885	0.3532
X_1_ × _3_	193.2800	1	193.2800	41.3000	0.0004**
X_2_ × _3_	0.4748	1	0.4748	0.1015	0.7594
X_1_^2^	406.1000	1	406.1000	86.7900	< 0.0001**
X_2_^2^	18.3300	1	18.3300	3.9200	0.0883*
X3^2^	431.1500	1	431.1500	92.1400	< 0.0001**
Residual error	32.7600	7	4.6800		
Lack of fit	11.7100	3	3.9000	0.7414	0.5804
Pure error	21.0500	4	5.2600		
Total variance	1310.6700	16			

*P is significant (*P* < 0.05), **P is extremely significant (*P* < 0.01). ANOVA = analysis of variance

### Calculation of the standard relation

The similarity between the reference sample obtained from the ancient decoction process and the samples obtained from the modern decoction process were determined using the standard relation method [5].

*Identification of the Subject of the Evaluation and Calculating the relative Deviation (RD*_*ij*_*).* The measured values of the reference sample were compared with the measured results values of the 17 experiments of the response surface experiment using the contents of the index components rosmarinic acid (Y_1_), isoimperatorin (Y_2_), puerarin (Y_3_), the extract yield (Y_4_), and fingerprint similarity (Y_5_) as the evaluation indexes. The RD_ij_ value of P_ij_ with respect to S_j_ was calculated using Eq. (1), where P_ij_ denotes the measured value under the jth (j = 1, 2, 3, …, n) metric of the ith (i = 1, 2, 3, …, m) sample of the Box-Behnken response surface experiment, and S_j_ denotes the measured value under the jth (j = 1, 2, 3, …, n) measured value under the index of the reference sample. An additional file shows this in more detail (see Additional file 2).

1 $${RD}_{ij}=\left|{P}_{ij}-{S}_{j}\right|/{S}_{j}$$

*Calculation of the standard Relation.* SR_ij_ denotes the standard relation under the jth (j = 1, 2, 3, …, n) metric for the ith (i = 1, 2, 3, …, m) sample of the Box-Behnken response surface experiment. The closer the value of SR_ij_ is to 100%, the closer the samples are to the reference sample. The SR_ij_ value is calculated by Eq. (2). An additional file shows this in more detail (see Additional file 2).

2 $${SR}_{ij}=1-{RD}_{ij}=1-\left|{P}_{ij}-{S}_{j}\right|/{S}_{j}$$

### Calculation of the comprehensive weights

*Subjective Weights of Fuzzy Analytic Hierarchy Process (FAHP).* FAHP is a subjective empowerment method that combines the analytic hierarchy process (AHP) and fuzzy mathematical theory to construct a fuzzy consistent judgment matrix, which facilitates results that are more in line with the human thinking mode in the case of more evaluation indexes, which are more fuzzy [6, 7]. First, a fuzzy judgment matrix was created. The index parameters of the extraction process were determined based on the theory of traditional Chinese medicine and the principle of ‘Sovereign, Minister, Assistant, and Guide’ in the compound prescription as the quality control means. As the fingerprint similarity provided limited information when comparing the content of indicator components, the final order of the index parameters was determined as rosmarinic acid (Y_1_) > isoimperatorin (Y_2_) > puerarin (Y_3_) > extract yield (Y_4_) > fingerprint similarity (Y_5_). According to the criteria for constructing the fuzzy judgment matrix (Table 5), the evaluation indicators were compared with each other so that subjective judgments and thought processes became quantitative and hierarchical, thus better reflecting the fuzziness of the problem and helping to obtain the fuzzy complementary judgment matrix (Eq. 3). The a_ij_ denotes the fuzzy relationship of the ith element of the lower layer relative to the jth element, and from this matrix, Eq. (4) can be obtained. Mathematical transformation of matrix A according to Eqs. (5) and (6) yields the fuzzy consistency judgment matrix (Eq. 7), which denotes the relative importance of the ith element to the jth element.

3 $$A={\left({a}_{ij}\right)}_{n\times n}=\left[\begin{array}{c}{a}_{11}{ a}_{12} \dots {a}_{1n}\\ {a}_{21} {a}_{22} \dots {a}_{2n}\\ \dots \dots \dots \dots \\ {a}_{n1} {a}_{n2} \dots {a}_{nn}\end{array}\right]$$

4 $${a}_{ij}+{a}_{ji}=1$$

5 $${r}_{i}={\sum }_{j=1}^{n}{a}_{ij}, \left(i=\text{1,2},\cdots ,n\right)$$

6 $${r}_{ij}=\frac{{r}_{i}-{r}_{j}}{2n}+0.5$$

7 $$R={\left({r}_{ij}\right)}_{n\times n}$$

The subjective weight coefficients of the evaluation indexes Y_1_, Y_2_, Y_3_, Y_4,_ and Y_5_ were calculated as 0.2650, 0.2350, 0.2000, 0.1750, and 0.1250, respectively, using Eqs. (8) and (9), and then, a consistency test was carried out. Assuming that W (Eq. 10) was the weight vector of the fuzzy consistency judgment matrix R, and W* (Eq. 11) was its feature matrix. When the consistency index $I\left(R,{W}^{*}\right)\le 0.1$, the judgment matrix could be categorised as a fuzzy consistency judgment matrix. The consistency test was passed by calculating I = 0.0380 < 0.1 using Eqs. (12) and (13).

8 $${w}_{i}=\frac{{\sum }_{k=1}^{n}{r}_{ik}+\frac{n}{2}-1}{n\left(n-1\right)}, \left(i=\text{1,2},\cdots ,n\right)$$

9 $${\sum }_{i=1}^{n}{w}_{i}=1, w>0, \left(i=\text{1,2},\cdots ,n\right)$$

10 $$W={\left({w}_{1},{w}_{2},\cdots ,{w}_{n}\right)}^{T}$$

11 $${W}^{*}={\left({w}_{ij}\right)}_{n\times n}$$

12 $${w}_{ij}=\frac{{w}_{i}}{{w}_{i}+{w}_{j}}, \left(\forall i,j=\text{1,2},\cdots n\right)$$

13 $$I\left(R,{W}^{*}\right)=\frac{1}{{n}^{2}}{\sum }_{i=1}^{n}{\sum }_{j=1}^{n}\left|{r}_{ij}+{w}_{ij}-1\right|\le a$$

*The Objective Weights of CRITIC.* Equation (14) was used to calculate the standard deviation α_j_, where j indicated the jth indicator and a indicated the ath indicator data; Eq. (15) was used to calculate the correlation coefficient β_j_, where m indicated the number of evaluation indicators, and r_ij_ indicated the correlation coefficient between evaluation indicators i and j. Equation (16) and Eq. (17) were used to obtain the final weights (${w}_{j}^{q}$). The CRITIC weights of Y_1_, Y_2_, Y_3_, Y_4_, and Y_5_ were calculated to be 0.2920, 0.3310, 0.1150, 0.2480, and 0.0140, respectively.

14 $${\alpha }_{j}=\sqrt{\frac{{\sum }_{a=1}^{n}{\left({x}_{aj}-{\stackrel{-}{x}}_{j}\right)}^{2}}{n-1}}$$

15 $${\beta }_{j}={\sum }_{i=1}^{m}\left(1-{r}_{ij}\right)$$

16 $${C}_{j}={\alpha }_{j}+{\beta }_{j}$$

17 $${w}_{j}^{q}=\frac{{C}_{j}}{{\sum }_{j=1}^{m}{C}_{j}}$$

*Calculation of the Comprehensive Weights.* Based on${ w}_{j}^{o}$obtained from FAHP and ${w}_{j}^{q}$ obtained from CRITIC, the comprehensive weight coefficients ${w}_{j}^{s}$ of each indicator are calculated according to formula (18) [8], and the comprehensive weights of Y_1_, Y_2_, Y_3_, Y_4_, and Y_5_ were calculated to be 0.3470, 0.3480, 0.1030, 0.1940, and 0.0080, respectively.

18 $${w}_{j}^{s}=\frac{{w}_{j}^{o}{w}_{j}^{q}}{{\sum }_{j=1}^{n}{w}_{j}^{o}{w}_{j}^{q}}$$

### Calculation of the comprehensive score

The comprehensive weight coefficients calculated from FAHP-CRITIC showed that Y_1_ accounted for 34.7%, Y_2_ accounted for 34.8%, Y_3_ accounted for 10.3%, Y_4_ accounted for 19.4%, and Y_5_ accounted for 0.8% in the optimization of STP extraction process. The Box-Behnken response surface experiment comprehensive scores were calculated according to the comprehensive score formula (19), and the results are shown in Table 5. The closer the comprehensive score was to 100, the closer was the quality of the samples produced by the process to that of the reference sample. The highest scores in the Box-Behnken response surface experiment were obtained for the group 15 experiments, so the optimal extraction process consisted of the addition of a volume of water 10 times that of the powder, an extraction time of 1 h, and 2 extractions.

19 $$\begin{aligned} M&={SR}_{i,{Y}_{1}}\times 0.347+{SR}_{i,{Y}_{2}}\times 0.348{+SR}_{i,{Y}_{3}}\times 0.103\\ &\quad+{SR}_{i,{Y}_{4}}\times 0.194{+SR}_{i,{Y}_{5}}\times 0.008\end{aligned}$$

**Table 5:** Criteria for FAHP to construct fuzzy judgment matrix

Scale	Definition	Explanation
0.1–0.4	In contrast	Comparing of the two indicators, ${a}_{ij}+{a}_{ji}=1$
0.5	Equal importance	Comparing the two indicators, both are equally important
0.6	Slightly important	Comparing the two indicators, the former is slightly more important than the latter
0.7	Clearly important	Comparing the two indicators, the former is clearly more important than the latter
0.8	Highly important	Comparing the two indicators, the former is considerably more important than the latter
0.9	Extremely important	Comparing of the two indicators, the former is extremely important compared to the latter

### Analysis of verification test

The process validation of the STP optimal extraction process obtained by screening through Box-Behnken response surface experiments was carried out in three parallel experiments to determine the content of rosmarinic acid (Y_1_), the content of isoimperatorin (Y_2_), the content of puerarin (Y_3_), the extract yield (Y_4_), the fingerprint similarity (Y_5_), and to calculate the comprehensive scores, and the results are shown in Table 6. The mean value of the comprehensive score obtained from the three batches of process validation experiments was 94.7, with an RSD value of 0.8%, indicating that the optimal extraction process of this STP was stable and highly reproducible.

**Table 6:** Results of response surface validation experiment

No.	Mass fraction (mg/g)			Y_4_ (%)	Y_5_	Comprehensive score
	Y_1_	Y_2_	Y_3_			
1	1.3231	0.0107	14.6278	25.8	0.9960	93.9
2	1.3304	0.0101	14.4465	24.7	0.9940	94.9
3	1.3247	0.0104	14.2997	26.0	0.9940	95.3
Mean value	1.3261	0.0104	14.3731	25.5	0.9950	94.7
RSD (%)	0.3	2.9	0.7	2.7	0.1	0.8

## Discussion

STP is a boiled powder formula. Due to limitations in equipment, energy consumption, efficiency, and other factors encountered during the actual production process, it is not feasible to fully replicate the traditional extraction process of boiled powder when developing innovative drugs. Therefore, this study refers to the requirements of the relevant technical guidelines for the development of ancient classic prescriptions, and based on the preliminary examination of the key information of the prescription, the reference samples were prepared according to the traditional process. These samples were compared with those prepared using the modern extraction process, and the standard relation was calculated to establish an interface between the ancient and modern processes in terms of quality.

Rosmarinic acid is a bioactive phenolic compound [9], which is one of the main active components in dried leaves of *Perilla frutescens* (L.) Britt. Further, rosmarinic acid has high antioxidant capacity [10–12], which is why it is a promising nutraceutical compound in the food industry. It exhibits antibacterial, antiviral, and anti-inflammatory [13–15] pharmacological activities, and it has considerable therapeutic potential in the treatment of cancer, diabetes, and neurodegenerative diseases [16–18]. Isoimperatorin has considerable potential for use in the treatment of lung diseases [19, 20]. Puerarin [21] is the major bioactive ingredient isolated from the root of the *Pueraria lobata* (Willd.) Ohwi, and it has neuroprotective, antioxidant, anticancer, and anti-inflammatory pharmacological activities [22–25]. When selecting the indicator components, the active ingredients in the sovereign medicinal should be selected first as the indicator parameters, while considering the stability of the components. The content of puerarin in the minister medicinal Gegen is high and stable, thus the process is based on the content of rosmarinic acid in the sovereign medicinal Zisuye and the content of isoimperatorin in Baizhi, as well as the content of puerarin in Gegen. However, using only the content of a few index components or extract yield as an evaluation index does not reflect the overall quality differences between samples. The fingerprint of Chinese materia medica reflects the contours and qualitative qualities of small-molecule components, and the method of controlling their quality through fingerprint similarity combined with quantitative analysis of the index components has been commonly used in the development of ancient formulas [26]. Therefore, in the present study, the fingerprint similarity between response surface test samples and reference samples was added as an index to evaluate consistency between ancient and modern extraction processes so that the optimised process would be more scientific and reliable.

FAHP is a subjective empowerment method based on AHP and fuzzy mathematical theory to solve the problem of consistency of hierarchical analysis [27–29]. The CRITIC method is an objective empowerment method using the objective attributes of data for scientific evaluation, which is a better method than the entropy weight and coefficient of variation methods [30]. In the study of extraction process optimization, single use of a certain assignment method ignores both the objective change law embodied in the data and the degree of subjective decision-making on different evaluation indexes. Therefore, the present study adopted the FAHP-CRITIC subjective-objective combination of empowerment methods to produce more reliable and accurate evaluation results.

Box-Behnken experimental design, standard relation, and FAHP-CRITIC Methods applied in the optimization of extraction processes are promising, especially in the study of ancient formulas with unique significance. The method can obtain stable and reliable extraction process parameters and provide data support for industrial production. The method can be applied to industrial production in the future. The process is adjusted and optimized according to the amplification results to obtain the most suitable process conditions for large-scale production and adapt it to the production demand so that products with high quality consistency with reference samples can be produced.

## Conclusion

In this study, a quality control system was established using rosmarinic acid, isoimperatorin, puerarin, extract yield, and fingerprint similarity as evaluation indexes of the extraction process. The optimal extraction process of STP was optimised by comparing the quality control standards of the reference samples, and the results of the validation experiments showed that the extraction process of STP was stable, reliable, and highly reproducible. Therefore, it is feasible to optimise the modern extraction process of ancient formulas using the standard relation combined with FAHP-CRITIC subjective and objective comprehensive weights, which provides an evaluation method ensuring consistency in the quality of samples produced using ancient and modern processes in current industrial production, as well as a theoretical basis for the study of modern extraction processes of other ancient formulas.

### Electronic supplementary material

Below is the link to the electronic supplementary material.

Supplementary Material 1

Supplementary Material 2

## Data Availability

The original contributions presented in the study are included in the article, and further inquiries can be directed to the corresponding authors.

## Abbreviations

AHP: Analytic hierarchy process
CRITIC: Criteria importance through intercriteria correlation
FAHP: Fuzzy analytic hierarchical process analysis
QbD: Quality by design
RSD: Relative standard deviation
SR: Standard relation
STP: Shenshou Taiyi Powder
TCM: Traditional Chinese medicine
